# Longitudinal Evidence on Peer Victimisation and Persistent Mental Health Outcomes in Youth: A Systematic Review

**DOI:** 10.3390/bs15121734

**Published:** 2025-12-15

**Authors:** Alessandra Giuliani, Irene Petruccelli, Giulio D’Urso

**Affiliations:** 1 Department of Psychology, University of Bath, Bath BA2 7AY, UK; ag3152@bath.ac.uk; 2 Faculty of Social and Communication Sciences, Universitas Mercatorum, 00186 Rome, Italy; 3 Department of Law, Economics, and Human Sciences, Mediterranea University of Reggio Calabria, 89124 Reggio Calabria, Italy

**Keywords:** peer victimisation, bullying, cyberbullying, longitudinal studies, child and adolescent mental health

## Abstract

Longitudinal studies consistently demonstrate that repeated exposure to peer victimisation can have enduring consequences for children and adolescents’ mental health. Documented outcomes include elevated risks of disorders such as depression, anxiety disorders, post-traumatic stress symptoms, and self-harm or suicidality, as well as broader psychosocial difficulties such as low self-esteem, loneliness, and internalising difficulties. However, prevalence estimates and effect sizes vary widely, reflecting differences in study design, measurement strategies, follow-up length, and the operationalisation of victimisation. This systematic review synthesises findings from 24 longitudinal studies to provide a comprehensive account of the mental health and psychosocial outcomes associated with peer victimisation. Evidence was strongest for depression and anxiety disorders, where multiple population-based cohorts in the United States, United Kingdom, Ireland, and Scandinavia showed robust prospective and retrospective associations, with risks amplified by repeated or persistent exposure. Peer victimisation also functioned as a traumatic stressor, predicting later post-traumatic stress symptoms, while self-harm and suicidality were elevated in several large-scale studies, though findings were less consistent. Additional work linked victimisation to later impaired well-being, including low self-esteem and internalizing difficulties. Mediators such as rumination, hostile attributions, and internalised stigma and moderators including gender, school belonging, and family support further explained heterogeneity in outcomes. By integrating findings across diverse contexts, this review clarifies the breadth and severity of long-term consequences of peer victimisation, highlighting both psychological mechanisms and contextual buffers. These insights can inform theoretical models of victimisation as a developmental risk factor and support the design of targeted prevention and intervention strategies.

## 1. Introduction

Mental health difficulties are a leading cause of disability worldwide, affecting approximately one in seven adults (Moitra et al., 2023). A growing body of research indicates that many of these difficulties have developmental origins, often traceable to early life experiences (Fryers & Brugha, 2013). Early prevention and intervention can substantially reduce the risk of later psychopathology (Colizzi et al., 2020). Among the most robust predictors of adverse outcomes is exposure to childhood adversity, which can disrupt normative trajectories of brain, behaviour, and emotion regulation across development (McLaughlin et al., 2019; Smith & Pollak, 2020).

Peer victimisation represents one particularly prevalent and impactful form of childhood adversity, with around one-third of children reporting past or current experiences (Craig et al., 2009). Peer victimisation is defined as intentional, repeated harm inflicted by peers within a context of power imbalance (Olweus & Limber, 2010) and is now recognised by the World Health Organization as a major global public-health issue (World Health Organization, 2018). Peer victimisation can take multiple forms (physical, verbal, relational, or cyber), each involving direct or indirect aggression intended to inflict harm (Shetgiri, 2013; J. Wang et al., 2009). Because of its repetitive, targeted, and intentional nature, peer victimisation extends beyond normative peer conflicts and poses a significant risk to children’s psychological well-being (Daley et al., 2023).

Victims of peer victimisation experience a broad spectrum of short- and long-term consequences across emotional, social, and physical domains (Catone et al., 2015). In the short term, children frequently exhibit heightened anxiety, social withdrawal, and vigilance to threat (Rivara et al., 2016). Over time, these difficulties can persist, contributing to poorer educational attainment, reduced socioeconomic status, physical illness, and interpersonal difficulties in adulthood (McDougall & Vaillancourt, 2015; Sigurdson et al., 2015). For example, adults who experienced bullying in childhood report higher rates of illness, poverty, and lower educational levels (Sigurdson et al., 2015). Victimisation has also been linked to criminal involvement, substance use, and intimate-partner violence (Klomek et al., 2010; B. Moore & Woodcock, 2017). Beyond these behavioural sequelae, growing evidence confirms the association between peer victimisation and long-term poor mental health (S. E. Moore et al., 2017). Youth exposed to peer victimisation are more than twice as likely to develop anxiety or depressive disorders a few years later (Ttofi et al., 2011). Even in later life, individuals who recall being bullied as children report higher depressive symptoms and lower life satisfaction (Peng et al., 2020). Although such retrospective studies are susceptible to recall bias, growing longitudinal evidence demonstrates associations between victimisation and depression, anxiety, self-harm, and suicidal ideation across diverse populations (Tsomokos & Slavich, 2024). Beyond its association with depression and anxiety, recent research increasingly conceptualises peer victimisation as a potentially traumatic experience that can generate symptoms similar to those seen following chronic interpersonal traumas (Idsoe et al., 2012). Peer victimisation may function as a repeated interpersonal threat that contributes to trauma-related symptomatology. While some authors conceptualise such experiences as “Type II trauma,” involving chronic and unpredictable exposure to harm (Terr, 1991) involving repeated, prolonged, and interpersonal stressors. However, we apply this framework cautiously, recognising that peer victimisation may fall outside traditional PTSD Criterion A definitions (American Psychiatric Association, 2013). Consistent with ICD-11, repeated interpersonal victimisation can nonetheless contribute to post-traumatic and complex trauma symptomatology, including disturbances in affect regulation, negative self-concept, and relational insecurity (World Health Organization, 2018). Consistent with this view, studies across school and workplace contexts have identified significant associations between victimisation and post-traumatic stress symptoms such as intrusive recollections, avoidance, hyperarousal, and emotional numbing across cohorts and sensitivity to different forms of victimisation (Hinduja & Patchin, 2025; Idsoe et al., 2012; Ossa et al., 2019).

Such long-lasting effects can be understood through several theoretical frameworks. Sensitive-period models posit that certain developmental windows are characterised by heightened neurobiological plasticity, during which environmental stressors have stronger and more persistent effects (Ho & King, 2021; Knudsen, 2004). Middle childhood and adolescence are periods of pronounced social sensitivity, during which peer relationships and social status become central to self-concept and emotional well-being (Somerville, 2013). Victimisation during these socially sensitive phases can therefore have enduring consequences for mental health. When conceptualised through trauma theory, repeated peer victimisation can affect coping and emotion-regulation systems, reinforcing difunctional reactions such as hypervigilance and perceived helplessness that resemble those seen in trauma-exposed individuals (Vasilopoulou et al., 2020; Zhao & Ye, 2025).

Biological perspectives offer complementary explanations for these effects. Chronic exposure to social threat can produce stress sensitisation, in which repeated activation of the hypothalamic–pituitary–adrenal axis leads to dysregulated cortisol responses and heightened emotional reactivity (Collip et al., 2011; Borrow et al., 2019). This dysregulation contributes to hypervigilance, negative self-appraisal, and threat perception bias, which in turn increases vulnerability to depression and anxiety (Murphy et al., 2022). From a trauma perspective, such physiological arousal parallels the intrusive memories and avoidance responses described in post-traumatic stress reactions (American Psychiatric Association, 2013), further supporting the conceptual overlap between chronic bullying and type II trauma. Over time, these biological and psychological alterations may consolidate maladaptive emotion-regulation patterns, creating a self-perpetuating risk for psychopathology.

Despite substantial research linking peer victimisation with adverse psychological outcomes, longitudinal evidence remains limited in clarifying when, how, and for whom these experiences translate into lasting psychological risk. Few longitudinal studies have systematically delineated the temporal and contextual conditions under which victimisation leads to lasting psychological risk, or identified the thresholds of duration, severity, and frequency that distinguish normative distress responses from clinically significant or chronic impairment. Furthermore, definitional inconsistencies and the exclusion of cyberbullying in many prior reviews limit the interpretability and generalisability of the findings (Griffin & Gross, 2004; S. E. Moore et al., 2017). Existing studies also vary widely in the outcomes assessed and in the extent to which moderators such as social support, resilience, or developmental timing are considered. Moreover, despite the established association between victimisation and mental health outcomes, these processes are not uniform across all victims. Individual, social, and contextual factors, such as emotion-regulation capacity, family cohesion, and broader ecological adversity, can moderate or mediate these outcomes (Coe et al., 2018; Cooley et al., 2022; S. Wang et al., 2025). Gender, for instance, is frequently identified as a moderator, with girls being more likely to exhibit internalising difficulties such as depression, anxiety, and general emotional distress, and boys showing more externalising or behavioural difficulties such as aggression or substance use (Lau et al., 2021; Ledwell & King, 2015). Yet findings remain inconsistent, suggesting that gender likely interacts with other moderators.

A comprehensive synthesis of longitudinal evidence is therefore needed to delineate which mental health and psychosocial outcomes are most consistently affected, and to identify the developmental and contextual conditions under which victimisation exerts its most enduring effects. The present systematic review aims to address these gaps by integrating longitudinal, quantitative evidence on the long-term mental health sequelae of peer victimisation in youth. By incorporating both traditional and digital forms of victimisation, this review examines the extent, persistence, and heterogeneity of outcomes, and highlights the moderators and mediators that may explain differential trajectories of adjustment following peer victimisation. Through this comprehensive synthesis, we aim to inform theoretical models of victimisation as a developmental risk factor and guide the design of more precise preventive and intervention strategies.

### Aims

Given the complexity and variability of the long-term consequences associated with peer victimisation, it is unlikely that any single study has captured the full spectrum of outcomes or the mechanisms that shape them. A systematic review, therefore, provides a means of bringing together diverse evidence to identify which mental health and psychosocial outcomes have been examined, and to assess under what conditions peer victimisation has the greatest impact. Unlike narrative accounts, a structured synthesis of longitudinal studies can highlight not only the persistence of effects but also the moderators and mediators that influence recovery or vulnerability.

The purpose of this review is thus to integrate findings from longitudinal quantitative research examining the effects of peer victimisation in childhood and adolescence. In particular, the review considers how victimisation characteristics, as well as features of the victims or their broader context, may explain heterogeneity in mental health outcomes. By doing so, we aim to provide a comprehensive picture of the breadth, severity, and persistence of mental health and psychosocial sequelae associated with peer victimisation in childhood and adolescence, and to identify where future preventive and supportive interventions might be most effective.

Specifically, we address the following research questions:

1. Which long-term mental health and psychosocial outcomes have been linked to peer victimisation in childhood and adolescence?
2. Which moderators and mediators account for variation in these associations across studies?
3. Which individual, social, and contextual factors are associated with variation in the likelihood or severity of later mental health difficulties following peer victimisation?

## 2. Methods

This review was conducted in accordance with the PRISMA (Preferred Reporting Items for Systematic Reviews and Meta-Analyses) guidelines (Page et al., 2021) and followed the methodological guidance outlined in the Joanna Briggs Institute (JBI) Manual for Evidence Synthesis (Aromataris et al., 2024). The review protocol was prepared a priori but not prospectively registered. In line with JBI recommendations for systematic reviews of aetiology and risk, we followed the following stages.

### 2.1. Protocol Development

An a priori protocol was prepared specifying the objectives, eligibility criteria, and planned analytical approach to ensure transparency and reproducibility.

### 2.2. Eligibility Criteria

Eligibility was defined a priori using the Population–Exposure–Outcome (PEO) framework (Aromataris et al., 2024).

The *population of interest* was children and adolescents who had experienced peer victimisation during childhood or adolescence, with the age range set at 5–25 years at the time of exposure. Studies including mixed-age samples were eligible only when results were disaggregated for this target group. The exposure age range reflects extended adolescence and emerging adulthood as key developmental stages for peer relationships and identity consolidation. Harmful peer dynamics frequently extend into post-secondary and workplace environments, including studies up to age 25, therefore allowing capture of victimisation occurring during socially sensitive developmental transitions.

The *exposure of interest* was peer bullying victimisation, including both traditional forms such as physical, verbal, and relational bullying, as well as cyberbullying.

The primary *outcomes of interest* were mental health outcomes assessed at least 3 months after exposure to peer victimisation. These encompassed clinical diagnoses made using recognised diagnostic criteria or structured diagnostic instruments, as well as symptoms of psychological distress reported in any form (e.g., validated scales or other systematically collected measures). Studies that reported behavioural consequences alone, such as aggression, substance use, or academic performance, were excluded. Where studies assessed outcomes at multiple follow-up points, preference was given to the longest available follow-up to capture enduring effects.

Eligible *study designs* included retrospective or prospective longitudinal studies with at least one follow-up assessment conducted at least 3 months after exposure to peer victimisation. Cross-sectional studies, narrative reviews, editorials, and commentaries were excluded. Only peer-reviewed journal articles were considered, and studies without an English-language abstract were excluded to ensure screening feasibility.

### 2.3. Search Strategy

First, an initial exploratory search was conducted to identify “seed” references and refine keywords and index terms. Building on this, a comprehensive search strategy was implemented on 17 September 2025 across the following databases: Scopus, PubMed, Embase, and ProQuest (ERIC, Australian Education Index, ProQuest Education, PsychInfo, and Premium collection in Sociology, Education and Social Sciences). No date or publication year restrictions were applied. Full search strings are reported in Appendix A.

### 2.4. Study Selection

All references obtained from the database searches were exported into Covidence (Veritas Health Innovation, Melbourne, Australia), which was used to manage screening and review decisions. The selection process was tracked on the platform. The first and last authors performed the screening.

**Figure 1:**
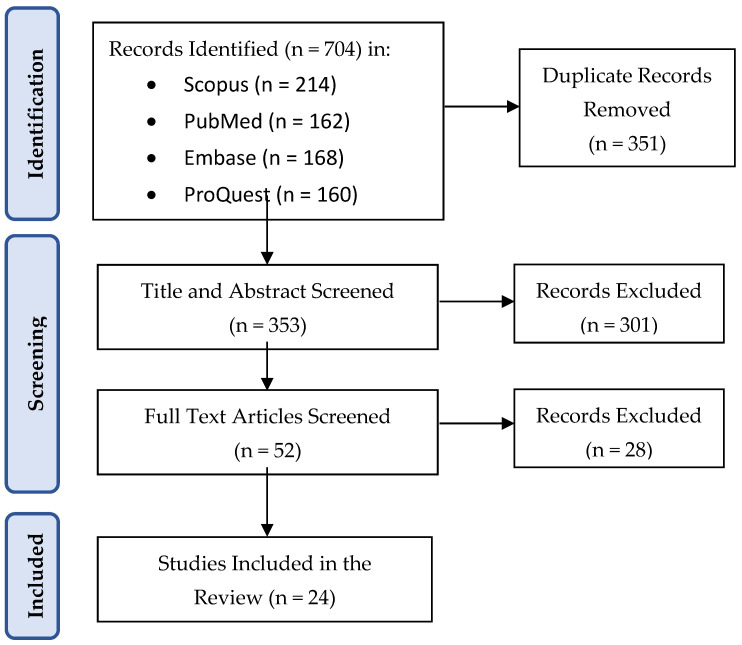
PRISMA flow diagram of identification, screening, and inclusion of records.

First, records from all databases were merged, and duplicates were removed. Title and abstract screening was then undertaken independently by two reviewers, using the predefined eligibility criteria. Each record was classified as “include,” “exclude,” or “maybe.” Across the 352 records screened, 37 conflicts were identified, resulting in an inter-rater agreement of 89.5% and a Cohen’s Kappa of 0.79 (95% CI: 0.73–0.85), reflecting substantial agreement (McHugh, 2012). Each disagreement was resolved through discussion. Citations marked as “include” or “maybe” were retrieved for full-text assessment. At this stage, each article was again reviewed independently by the two researchers and classified as “include” or “exclude”. During full-text screening, 6 conflicts arose out of 52 records, yielding 88.5% agreement and a Cohen’s Kappa of 0.77 (95% CI: 0.60–0.94). Reasons for exclusion were recorded in detail, such as studies reporting only prevalence without longitudinal follow-up, those restricted to behavioural or academic outcomes rather than mental health, and those including mixed-age samples without separate reporting for children or adolescents. Articles meeting all criteria were added to the final pool of included studies. The number of records identified, screened, excluded, and retained is reported in the PRISMA flow diagram (Figure 1). All the included studies are outlined in Table 1.

### 2.5. Certainty of Evidence

We considered applying the GRADE framework (Guyatt et al., 2008); however, due to substantial heterogeneity in exposures, outcomes, follow-up windows, and analytical strategies across studies, aggregated certainty ratings were deemed inappropriate. Instead, we used CASP (Critical Appraisal Skills Programme, 2024) Cohort Study Checklist, which is appropriate for evaluating longitudinal observational research, to appraise study quality and directly integrated risk-of-bias considerations into the interpretation of findings. For each checklist item, the maximum possible score was 12, reflecting the number of relevant CASP questions for cohort studies. Studies achieving scores between 9 and 12 were classified as high quality, those scoring 6 and 8 as moderate quality, and those scoring 0 and 5 as low quality. The distribution of quality ratings across the evidence base is presented in Table 1. This structured appraisal was used to inform the synthesis and interpretation of results.

### 2.6. Data Extraction and Synthesis

A standardised extraction form, developed a priori and piloted on a subset of studies, was used to ensure consistency and transparency in data collection. The following information was recorded for each study: study identifiers (authors, year, journal); country/setting; design; sample size; participant characteristics (mean age, age range, sex distribution); peer victimisation exposure definition, instrument and informant (self/peer/teacher), type (physical/verbal/relational/cyber); mental health outcome; timing of assessment of the outcome; statistical analysis; effect estimates (unadjusted and adjusted odds ratios [OR], risk ratios [RR], hazard ratios [HR], regression coefficients, mean differences) with 95% confidence intervals; covariates in adjusted models; mediators/moderators where present and their effect estimates. Extracted effect estimates prioritised models adjusted for core confounders when available. Data were synthesised in a tabular and narrative structure by key mental health outcomes, while also considering the type and measurement of victimisation and follow-up length. Within this framework, we compared studies reporting on moderators and mediators. References related to SR are marked with an asterisk in the final reference list.

## 3. Results

### 3.1. Quality Appraisal

All studies were rated between 9 and 12 on the CASP checklist, indicating high overall quality. However, several common limitations were identified across many of the 24 studies. The most significant of these was the over-reliance on self-report measures of victimisation and outcomes. Although these measures were typically validated or had high Cronbach’s alpha values, and some studies utilised a multi-informant approach, the risk of bias remains. Additionally, over half of the studies had follow-up periods of less than two years, which may limit their ability to fully capture the long-term effects of victimisation on mental health outcomes. Although many studies controlled for baseline symptoms, several did not adequately account for family- or school-level confounding, increasing risk of bias through residual confounding. Finally, many studies were conducted within a single country and culture (with most taking place in European and American countries), reducing the studies’ ecological validity and generalisability of the results. These issues likely contribute to heterogeneity across studies and should temper the strength of conclusions drawn from the current evidence base. Due to substantial heterogeneity in measurement tools, outcome constructs, reporting formats, and timing of follow-up assessments, quantitative pooling of effect sizes was not feasible. Consequently, we were unable to examine heterogeneity or provide meta-analytic estimates of associations statistically and instead report effect sizes narratively where available. Effect sizes and confidence intervals were reported where available; however, inconsistency in how these metrics were presented across studies limited comparability. The synthesis is shown in Table 2.

### 3.2. Studies Characteristics

The 24 included studies were predominantly prospective longitudinal cohorts, with follow-up periods ranging from a few months to up to roughly a decade (e.g., Copeland et al., 2013; Isaacs et al., 2008; Lereya et al., 2015). Only a few studies adopted a retrospective design, asking adults to recall whether they had been bullied during childhood or adolescence (e.g., Espelage et al., 2016; Lund et al., 2009). Most were conducted in Europe and North America, though some work was based in Africa (Boyes et al., 2014; Lee et al., 2024) and focused on clinical populations such as adolescents living with HIV (Boyes et al., 2020), children with ADHD (Orengul et al., 2023), or youth with chronic pain (Nania et al., 2024). Sample sizes varied widely, ranging from small clinical or school-based cohorts (between 70 and 400 participants; e.g., Cosma et al., 2018; Wright & Wachs, 2019) to large national or population-based studies (over 6000 participants; e.g., Lund et al., 2009). Participants were mostly children and adolescents, with outcomes often tracked into young adulthood (Due et al., 2009; Winding et al., 2020) or, in some cases, midlife (Lund et al., 2009).

Exposure to peer victimisation was assessed with diverse instruments: most commonly self-report questionnaires (from single-item frequency questions to multi-item scales capturing physical, verbal/relational, and cyber forms) (e.g., Liu et al., 2020; Manrique et al., 2020; Winding et al., 2020; Wright & Wachs, 2019); peer nominations identifying classmates frequently targeted (Isaacs et al., 2008; Perren et al., 2013); multi-informant teacher/parent reports combined with child ratings (Rudolph et al., 2011); and interviewer-administered modules embedded in structured assessments or surveys (e.g., CAPA/YAPA-style interviews and JVQ-based telephone surveys) (Copeland et al., 2013; Turner et al., 2020; Lee et al., 2024). Mental health outcomes were diverse but clustered around internalising symptoms such as depression and anxiety (Boyes et al., 2014; Due et al., 2009), post-traumatic stress symptoms (Turner et al., 2020; Liu et al., 2020), or adult psychiatric diagnoses (Copeland et al., 2013). A few studies also examined protective or mediating factors, such as family support (Isaacs et al., 2008), school belongingness (Wright & Wachs, 2019), or rumination (Liu et al., 2020). Analytically, most studies employed multivariable regression or structural equation modelling to test associations.

### 3.3. Depression

In total, 17 studies assessed depression as a long-term consequence of victimisation. Some studies measured it as clinical depressive disorder (e.g., Copeland et al., 2013; Lereya et al., 2015), others as symptom scales (e.g., Boyes et al., 2020; Lee et al., 2024; Wright & Wachs, 2019), and others included it as part of internalising composites (e.g., Boyes et al., 2014; Cosma et al., 2018). Across cohorts and contexts, peer victimisation was prospectively associated with higher depressive symptoms. In the United States and the United Kingdom, exposure to bullying in childhood/adolescence predicted depressive disorders in young adulthood, even after controlling for baseline psychopathology and family adversity, and effects scaled with persistence of exposure (Copeland et al., 2013; Lereya et al., 2015). Long-horizon European cohorts corroborated this pattern: in Denmark, being bullied at one vs. two adolescent time-points showed a dose–response increase in depression risk at age 28; while a historical cohort of Danish men indicated elevated probability of current and past diagnosis of depression in adulthood among those frequently bullied in adolescence (Lund et al., 2009; Winding et al., 2020). Developmentally, U.S. latent-growth work showed that both higher early levels and increases in victimisation across elementary school predicted greater depressive symptoms by Grade 5, underscoring that initial exposure and worsening trajectories each confer risk (Rudolph et al., 2011). In a Sub-Saharan African cohort, victimisation was also linked to higher depressive symptom counts at one-year follow-up after adjustment for baseline mental health and socioeconomic covariates, expanding generalisability beyond high-income settings (Lee et al., 2024).

Not all studies detected significant prospective effects after accounting for confounding and baseline symptoms. In South African community samples, peer victimisation showed small, non-significant prospective effects on subsequent depressive symptoms after adjustment (Boyes et al., 2014). Among adolescents living with HIV, the adjusted paths to depression at 18 months were also non-significant (β = 0.05, *p* = 0.165), suggesting that structural and health-related stressors may overshadow the unique contribution of peer victimisation in high-adversity clinical populations (Boyes et al., 2020). Null or weak prospective paths also appeared in some European school cohorts that modelled cross-lagged internalising composites (Cosma et al., 2018), and a follow-up in Turkey reported only marginal prediction of internalising difficulties at one year (Orengul et al., 2023).

Cybervictimisation was also a consistent short-term predictor of depressive symptoms in adolescent cohorts after controlling for baseline depression and face-to-face bullying, with social context shaping the size of effects (Wright & Wachs, 2019). In U.S. middle schools, cybervictimisation predicted higher depression one year later, with stronger associations among students reporting low school-belongingness and among Latinx adolescents (Wright & Wachs, 2019). Among LGBTQIA adolescents, homophobic cybervictimisation (and bystanding) predicted higher depressive symptoms at one year. However, perceived support from school-based Gay–Straight Alliances buffered these effects, indicating modifiable contextual protection (Wright et al., 2022). Cross-sectional college data aligned with these longitudinal signals, linking recalled peer victimisation to current depressive symptoms alongside anxiety and PTSD (Espelage et al., 2016), while U.S. criminological work tying repeated pre-12 bullying to poorer mental health provides convergent evidence that cumulative exposure matters (Bouffard & Koeppel, 2014). Socioeconomic context also conditioned risk: in a large European study, the association between bullying and depression was stronger among young people with lower socio-economic status (Due et al., 2009). Swedish registry-linked analyses further suggested sex-specific timing and persistence effects: bullying in Grade 11 and sustained exposure from Grades 9–11 predicted depression by age 20–21 in females, whereas patterns were weaker or imprecise in males (Låftman et al., 2024).

Several studies explored how victimisation becomes embedded as depressive symptomatology through mediation and moderation analyses. In Chinese adolescents, intrusive (and to a lesser extent deliberate) rumination mediated the link between cybervictimisation and later depression, pointing to maladaptive cognitive processing as a pathway of risk (Liu et al., 2020). Swiss longitudinal SEM work indicated that hostile attribution biases and self-blame intensified internalising pathways, with self-blame interacting with victimisation to amplify later symptoms (Perren et al., 2013). In U.S. college samples, perceived social support partially mediated associations between multiple forms of prior peer victimisation and current depressive symptoms (Manrique et al., 2020). On the other side, protective environments moderated risk of developing depression symptoms: school-belongingness and Gay–Straight Alliance support weakened cyber-related consequences (Wright & Wachs, 2019; Wright et al., 2022), socioeconomic disadvantage strengthened them (Due et al., 2009), and family support attenuated longer-term links between adolescent victimisation and adult self-evaluations that track closely with depressive affect (Isaacs et al., 2008).

### 3.4. Anxiety

In total, 10 studies investigated whether anxiety was a long-term consequence of peer victimisation in youth. Across these, anxiety was assessed either as DSM-based diagnoses in young adulthood or as validated symptom scales in adolescence. In the Great Smoky Mountains Study in the USA, childhood/adolescent bullying predicted young-adult DSM anxiety disorders (including panic disorder, agoraphobia, and generalised anxiety) after rigorous adjustment, underscoring disorder-level risk (Copeland et al., 2013). A parallel pattern was observed in the UK, where bullied adolescents showed elevated anxiety symptoms at age 18, even after controlling for early internalising difficulties and sociodemographic factors (Lereya et al., 2015). Registry-linked analyses from Sweden added developmental nuance: grade-9 bullying predicted later anxiety in boys, whereas grade-11 and persistent bullying predicted anxiety in girls, indicating sex- and timing-sensitive risk (Låftman et al., 2024).

Cyber contexts replicated and extended these findings. In a U.S. middle-school cohort, baseline cybervictimisation predicted higher symptoms of anxiety one year later, independent of baseline anxiety and face-to-face bullying; effects were amplified under low school-belongingness and were stronger among Latinx youth (Wright & Wachs, 2019). Among LGBTQIA adolescents, homophobic cybervictimisation and bystanding prospectively increased anxiety, while perceived Gay–Straight Alliance support buffered these associations (Wright et al., 2022). School-based work separating physical, verbal, and social victimisation found each subtype associated with higher anxiety symptoms in hierarchical models, again using validated anxiety scales (Manrique et al., 2020). Retrospective studies also found recalled peer victimisation to be associated with current elevated anxiety symptoms (Espelage et al., 2016).

On the other hand, in South African cohorts, adjusted prospective paths from peer victimisation to anxiety over 18 months were small and non-significant among adolescents living with HIV (β = 0.01, *p* = 0.780), and earlier community-based work reported weak internalising paths once baseline symptoms were controlled for (Boyes et al., 2020; Boyes et al., 2014). A Romanian three-wave school study modelling internalising composites also did not detect significant cross-lagged effects over 6–12 months (Cosma et al., 2018). Finally, a small controlled follow-up in Turkey reported anxiety but lacked fully adjusted prospective estimates for that outcome (Orengul et al., 2023).

Evidence on mediators and moderators of the association between victimisation and anxiety is more limited. In U.S. school data, perceived social support did not significantly mediate the association between victimisation and anxiety, even though it mediated depression/PTSD in the same analyses, suggesting outcome-specific pathways (Manrique et al., 2020). By contrast, contextual moderators were consistent: low school-belongingness strengthened the associations between cyberbullying and anxiety, with a significant three-way interaction indicating stronger effects for Latinx youth under low belongingness (Wright & Wachs, 2019), and higher gay-straight alliance support buffered anxiety of homophobic cybervictimisation/bystanding in LGBTQIA adolescents (Wright et al., 2022). In high-adversity clinical samples, internalised HIV stigma emerged as a small but significant mediator of higher anxiety, helping to explain why the direct victimisation paths were null once broader stressors were considered (Boyes et al., 2020).

### 3.5. Post-Traumatic Stress and Trauma Symptoms

In total, 8 studies explored the association between youth victimisation and traumatic symptoms, finding that bullying experiences can act as traumatic stressors, contributing to post-traumatic stress symptoms (PTSS) and, in some cases, diagnosed post-traumatic stress disorder (PTSD). In a U.S. nationally representative cohort, repeated victimisation, particularly physical intimidation, emotional bullying, and internet harassment, predicted elevated trauma symptoms two years later, highlighting the cumulative burden of exposure (Turner et al., 2020). Findings from Burkina Faso further confirmed that peer victimisation predicted elevated PTSS risk even after controlling for demographic and family-level factors (Lee et al., 2024). Evidence from cybervictimisation studies converges on similar conclusions. In Chinese adolescents, cyberbullying predicted PTSS both directly and indirectly through intrusive rumination, suggesting that maladaptive cognitive processing may sustain trauma responses (Liu et al., 2020). Likewise, in a clinical cohort of youth with chronic pain, relational victimisation was associated with greater pain interference three months later, and this effect was fully mediated by PTSS (Nania et al., 2024).

Moderation analyses extend these findings by highlighting the importance of social context. In U.S. middle-school students, cybervictimisation predicted trauma symptoms more strongly under conditions of low school belongingness and among Latinx adolescents (Wright & Wachs, 2019). Among LGBTQIA youth, perceived support from Gay–Straight Alliances buffered the negative impact of homophobic cybervictimisation, attenuating trajectories towards both anxiety and PTSS (Wright et al., 2022).

### 3.6. Suicidality and Self-Harm

Five longitudinal studies investigated the relationship between peer victimisation and subsequent self-harm or suicidality. In the USA Great Smoky Mountains Study, victimised children were more likely to meet criteria for suicidality in young adulthood, although the effect was weaker than for depression or anxiety and did not always reach significance after full adjustment for baseline psychopathology and family adversity (Copeland et al., 2013). Stronger findings emerged in larger population-based cohorts, where victimisation predicted higher odds of both self-harm and suicidality at follow-up (Lereya et al., 2015). Evidence from the West Jutland cohort in Denmark also reinforced these results, showing that adolescents who were bullied at one or more time points were at progressively higher risk of reporting suicidal ideation and self-harm in adulthood (Winding et al., 2020). In Burkina Faso, peer victimisation significantly predicted self-harm over a one-year follow-up, with gender moderating effects showing that victimised girls were more vulnerable than victimised boys (Lee et al., 2024). Finally, in a USA study linking self-esteem, depression, and suicidality, peer victimisation predicted lower self-esteem and higher depression, which in turn increased risk of suicidality, suggesting an indirect pathway through internalising difficulties (Isaacs et al., 2008).

### 3.7. Internalising Difficulties, Self-Esteem, and Loneliness

Seven studies assessed broader internalising difficulties as outcomes of peer victimisation. In Romania, a study on 102 secondary school students found no significant effects of victimisation on subsequent internalising problems at 6 and 12 months (Cosma et al., 2018). In Turkey, a one-year follow-up showed marginal associations between victimisation and internalising symptoms (Orengul et al., 2023). By contrast, evidence from Switzerland demonstrated that peer-nominated victimisation predicted increased internalising difficulties over two years, with hostile attributions and self-blame significantly mediating these effects, highlighting the role of maladaptive cognitive processing (Perren et al., 2013). South African data provided mixed results: Boyes et al. (2014) reported a small but significant prospective effect of victimisation on internalising problems after adjustment for baseline symptoms, although subsequent analyses in adolescents living with HIV failed to replicate this (Boyes et al., 2020). More recently, the Growing Up in Ireland cohort showed that peer victimisation at age 13 predicted emotional problems at age 17, with emotional difficulties persisting into age 20 and effects robust to adjustment for gender, social class, and cognitive ability (D’Urso et al., 2024). In Finland, a study by Isaacs et al. (2008) showed that victimisation predicted lower self-esteem. In U.S. middle-school students, cybervictimisation was a significant predictor of loneliness at one-year follow-up, with effects exacerbated by low school belongingness (Wright & Wachs, 2019).

## 4. Discussion

This systematic review synthesised longitudinal evidence on the long-term mental health consequences of peer victimisation in youth. Across the 24 studies included, peer victimisation consistently emerged as a robust developmental risk factor for multiple domains of psychopathology, including depression, anxiety, post-traumatic stress symptoms, self-harm and suicidality, and broader internalising and psychosocial difficulties. Importantly, our synthesis demonstrates that both traditional face-to-face bullying and cybervictimisation are associated with enduring mental health outcomes. Across the evidence base, findings converged in showing that the persistence, chronicity, and relational or identity-targeted nature of victimisation are particularly associated with long-term psychological consequences. However, there was also variability in effect sizes, and several studies reported null or attenuated findings, indicating that adverse outcomes are not universal and may depend on co-occurring risks and protective resources. It is worth noting that although the longitudinal nature of the included studies supports temporal ordering, these associations cannot establish causality; unmeasured confounding, bidirectionality, and shared-method variance remain plausible alternative explanations.

This body of evidence highlights both the robustness of peer victimisation as a predictor of later maladjustment and the methodological complexity that underpins variability in effect sizes across studies. In particular, heterogeneity in peer victimisation exposure measurement, outcome definition, and follow-up duration limited comparability and likely contributed to the range of reported effect sizes. Null or attenuated findings in some high-adversity samples also underscore that victimisation does not act in isolation but interacts with broader contextual factors. Taken together, these findings reinforce the need to conceptualise peer victimisation as a correlational developmental risk indicator for later mental health difficulties, acknowledging that observed associations may reflect indirect or reciprocal processes. We discuss the evidence for specific outcomes in turn below. The convergence of results across diverse cultural and socioeconomic settings supports the conceptualisation of peer victimisation as a form of chronic interpersonal stressor. Consistent with trauma theory (Idsoe et al., 2012), repeated exposure to social threat may function as a Type II trauma (Van der Kolk, 2005), disrupting cognitive-emotional and physiological regulation systems. Longitudinal findings linking persistent or identity-based victimisation to depressive and anxiety trajectories align with models of stress sensitisation (Bunea et al., 2017; Miller et al., 2025), suggesting that recurrent activation of the hypothalamic–pituitary–adrenal axis leads to dysregulated stress reactivity and heightened emotional responsivity to later adversity. From a developmental neuroscience perspective, these experiences likely interact with sensitive periods of social and neural plasticity (Knudsen, 2004; Ho & King, 2021), during which repeated rejection, exclusion, or humiliation can have disproportionate and enduring effects on emotion regulation, social cognition, and self-concept formation.

### 4.1. Disorder-Specific Pathways

Our review identified several domains of psychopathology that were consistently predicted by exposure to peer victimisation. Depression emerged as the most robust and frequently investigated outcome. Across large-scale longitudinal studies such as ALSPAC and the Great Smoky Mountains Study, victims of bullying were significantly more likely to develop depressive symptoms and major depressive disorder in adolescence and young adulthood, even when early emotional problems and family adversity were controlled for (Copeland et al., 2013; Lereya et al., 2015). These findings were replicated in Scandinavian cohorts, where adolescents repeatedly exposed to bullying between ages 15 and 18 were more than twice as likely to present with depression in adulthood (Lund et al., 2009; Winding et al., 2020). Similar trajectories were also found in lower-income contexts, including Burkina Faso (Lee et al., 2024), suggesting that the psychological consequences of victimisation generalise across cultural and economic settings. However, the absence of significant effects in two South African studies (Boyes et al., 2014, 2020) highlights that contextual adversities may soften the specific contribution of peer victimisation to later depression.

The overall picture indicates that depressive outcomes are particularly likely when victimisation is chronic, relational, or identity-targeted. In these cases, experiences of exclusion and humiliation may negatively affect self-esteem and produce persistent negative self-schemas. Supporting this interpretation, several of the included studies found that self-blame, intrusive rumination, hostile attributions, and internalised stigma mediated the link between bullying and later depression (Liu et al., 2020; Perren et al., 2013). In contrast, supportive relational contexts, particularly high family cohesion and school belonging, were shown to attenuate the negative effects of victimisation (Isaacs et al., 2008; Wright & Wachs, 2019).

Anxiety outcomes followed a similar pattern of risk, with peer victimisation predicting elevated generalised anxiety, panic, and phobic symptoms later in time. The Great Smoky Mountains and ALSPAC cohorts both reported strong associations between early bullying and later anxiety disorders (Copeland et al., 2013; Lereya et al., 2015), while more recent population-based research in Sweden revealed gendered trajectories: early-onset victimisation increased anxiety particularly among boys, whereas persistent victimisation exposure had stronger effects for girls (Låftman et al., 2024). The rise of digital media has expanded this literature, showing that cybervictimisation produces comparable or even stronger anxiety effects than face-to-face bullying. Among U.S. middle school students, cybervictimisation predicted heightened anxiety one year later, even when baseline anxiety and traditional bullying were accounted for, with the strongest effects observed among students with low school belongingness and social connectedness (Wright & Wachs, 2019). Among LGBTQIA youth, homophobic cyberbullying and bystanding predicted anxiety increases, though support from Gay–Straight Alliances buffered these effects, suggesting that inclusive school climates can meaningfully moderate victimisation risk (Wright et al., 2022).

Beyond mood and anxiety, several studies analysed peer victimisation as a traumatic stressor. Nationally representative data from the United States indicated that new and repeated victimisation was prospectively associated with elevated trauma symptoms (Turner et al., 2020). In Burkina Faso, sexual and peer victimisation both predicted PTSD severity, with independent effects after demographic and familial adjustment (Lee et al., 2024). Cognitive pathways again played a critical role, with intrusive rumination and avoidance behaviours shown to mediate the relationship between cybervictimisation and PTSS (Liu et al., 2020), while among youth with chronic pain, PTSS fully mediated the association between relational victimisation and pain interference (Nania et al., 2024). These findings collectively indicate that bullying, particularly when repeated or relational, may act as a type of interpersonal trauma, potentially triggering post-traumatic stress reactions such as hyperarousal, intrusive thoughts, and emotional numbing.

Finally, evidence regarding self-harm and suicidality was less consistent but pointed toward a meaningful association. In the ALSPAC and GSMS cohorts, victimisation during adolescence significantly increased the risk of self-harm and suicidal ideation into young adulthood (Lereya et al., 2015). Similar effects were observed in the West Jutland cohort, where persistent bullying exposure predicted substantially higher odds of suicidality (Winding et al., 2020). However, studies with more limited or single-item measures reported weaker or null findings (Copeland et al., 2013). Suicidality appears to operate as a downstream consequence of victimisation-driven depression and low self-worth (Isaacs et al., 2008), with gender emerging as an important moderator, as girls, particularly in low-resource settings, showed greater vulnerability to it (Lee et al., 2024).

Taken together, the evidence positions peer victimisation as a major determinant of youth mental health, with consequences that often persist beyond youth. Rather than a transient social challenge, peer victimisation constitutes a chronic interpersonal stressor which shapes emotional, cognitive, and relational development. Accordingly, to address this, prevention and intervention strategies should ensure that schools, health services, and policy frameworks work together to identify and support affected young people. At the educational level, school programmes should focus on developing empathy, emotion regulation, and inclusive peer cultures, factors shown to buffer the psychological sequelae of peer victimisation (Wright & Wachs, 2019; Låftman et al., 2024). For clinicians, systematic assessment of victimisation histories should be standard practice, with therapeutic approaches targeting maladaptive processes such as rumination, self-blame, and internalised stigma, while strengthening self-compassion and regulatory capacities. Given the differential patterns observed across genders, interventions should also be tailored to address these distinct manifestations of distress and coping. Sustained policy investment in safe school climates and early-intervention infrastructures is therefore essential. By recognising bullying as a public-health and developmental risk, not merely a social conflict or a transient challenge, we can advance more integrated and enduring approaches to lifelong mental-health prevention and care. While the reviewed studies consistently demonstrated elevated mental health risks among victimised youth, outcomes varied across gender, cultural context, and adversity exposure. A clearer understanding of when and for whom victimisation is most harmful will be essential for informing targeted prevention and support strategies.

### 4.2. Limitations and Future Directions

Despite the strengths of this systematic review, some important limitations need to be highlighted. First, we restricted inclusion to peer-reviewed journal articles and excluded grey literature (e.g., theses, dissertations, books, non-peer-reviewed reports). While this decision enhanced methodological consistency, it may have reduced coverage and introduced publication bias toward studies with statistically significant results. The evidence base may also be affected by publication bias. Studies reporting non-significant or null findings, particularly in high-adversity samples where competing stressors may overshadow bullying effects, are less likely to be published. This is supported by several included studies reporting small or null prospective associations (e.g., Boyes et al., 2014, 2020). Therefore, overall effect sizes may be inflated due to selective publication. Additionally, as only English-language articles were eligible for inclusion, studies published in other languages may be underrepresented. This language restriction may have contributed to a geographical bias toward high-income, English-speaking countries and could limit the global generalisability of the findings.

A second limitation is the marked heterogeneity in outcome measurement. While some studies assessed clinically diagnosed disorders, using dichotomous categories, others relied on continuous symptom scales. Diagnostic thresholds are likely to underestimate the mental health consequences, as they exclude individuals with subclinical symptomatology. Equally, victimisation exposure was assessed through diverse approaches, including single items, multi-item self-reports, peer nominations, teacher or parent reports, and structured psychiatric interviews. Each method differs in its sensitivity to covert or relational forms of bullying and in its susceptibility to recall or informant bias. In addition, definitions and thresholds for victimisation (e.g., frequency, severity, inclusion of cyber forms) were inconsistent across studies, reducing comparability and contributing to heterogeneity in reported associations. Such variability, compounded by differences in follow-up length, analytic strategy, and covariate adjustment, likely explains some of the inconsistencies and null findings across the literature. Moreover, over 70% of studies relied solely on self-report, which increases the risk of recall and perceptual bias and potentially inflating effect sizes due to shared-method variance. To address this, future research should focus on developing harmonised, multi-informant measures of peer victimisation and mental health outcomes, ensuring comparability across cohorts and sensitivity to different forms of victimisation.

Another important consideration is that most studies adjusted for baseline mental health. While this is a strength, it may also introduce challenges: if prior symptoms lie on the causal pathway (e.g., early distress increasing risk of victimisation), such adjustment may attenuate true effects. More broadly, because all included studies were observational, causality cannot be inferred; it remains possible that early internalising difficulties increase both risk of victimisation and later symptoms, contributing to recursive or bidirectional effects over time. In addition, over half of the included studies followed participants for up to two years, limiting conclusions about longer-term trajectories. Finally, our review deliberately focused on the long-term consequences of peer victimisation (at least 6 months after exposure to victimisation), which means that short-term consequences were outside our scope. Firstly, this may bias estimates toward healthier survivors. Secondly, short-term mental health responses may play a crucial role in shaping subsequent trajectories. For example, early distress could mediate pathways to chronic psychopathology or contribute to academic and social difficulties that reinforce later risk. Future research would therefore benefit from a dual focus, examining both short-term and long-term consequences in parallel, ideally within the same cohorts, to clarify how early responses to victimisation consolidate into persistent mental health problems. Finally, although an a priori protocol guided study aims, eligibility criteria, and planned synthesis, the review was not prospectively registered. This increases the risk of selective reporting and analytic flexibility, and future reviews in this field should register protocols to strengthen transparency and reproducibility.

## 5. Conclusions

This review systematically evaluated the long-term mental health outcomes associated with peer victimisation during childhood and adolescence. The findings across the included longitudinal studies were largely consistent, indicating that both face-to-face and cyber forms of victimisation are significant predictors of later psychological difficulties. The most consistent and well-supported evidence concerned depressive outcomes, followed by anxiety and post-traumatic stress symptoms. Evidence for broader internalising problems, such as general emotional distress or reduced self-esteem, was present but less extensive and more variable across samples and contexts. Overall, these findings confirm that exposure to peer victimisation in youth is not a transient social experience but a meaningful developmental stressor with serious consequences for mental health over time.

Importantly, the evidence reviewed also indicated that contextual protective factors, such as supportive family relationships and inclusive school environments, may substantially attenuate these mental health consequences. This highlights a need for greater attention to the mechanisms that explain how these outcomes occur. Understanding these mechanisms is essential to inform targeted interventions aimed at reducing the long-term psychological consequences of victimisation once it has occurred. However, while developing interventions to mitigate harm is essential, this should not divert attention from aiming at preventing peer victimisation in the first place. The consistent demonstration of its adverse and lasting mental health consequences underscores the importance of effective victimisation prevention strategies.

## Figures and Tables

**Table 1:** Overview of Included Studies.

Study	Location	*n* and Gender Proportion	Age Range at Victimisation Exposure	Data Collection Approach	Analytical Method	CASP Rating
Bouffard and Koeppel (2014)	USA	*n* = 7898 (51.4% female, 50.1% male)	12–14 years	Interview data from six waves of NLSY	Regression	11
Boyes et al. (2014)	South Africa (Western Cape and Mpumalanga)	*n* = 3515 (57% female, 43% males)	10–17 years	Door-to-door household sampling; interviewer-administered questionnaires	ANCOVA; Cross-Lagged SEM; Regression	9.5
Boyes et al. (2020)	South Africa (Eastern Cape)	*n* = 1060 (45% males, 55% females)	10–19 years	Structured interviews and validated scales	Regression; Mediation	10
Copeland et al. (2013)	USA (Western North Carolina)	*n* = 1420 (49% female, 51% male)	9–16 years	Structured interviews with parents and self	Regression	11
Cosma et al. (2018)	Romania (Cluj-Napoca and Floresti)	Final *n* = 102 Baseline *n* = 185 (47.8% male, 52.2% female)	12–16 years	Self-report questionnaires	Cross-Lagged Panel Modelling	10
Due et al. (2009)	Denmark	*n* = 614 (60.3% female, 39.7% male)	15 years	Self-report anonymous postal surveys (1990 baseline, 2002 follow-up)	Analyses of Variance (ANOVA and MANOVA); Interaction Analyses	11.5
D’Urso et al. (2024)	Ireland	*n* = 7525 (51.1% female, 48.9% male)	13 years	Multi-informant (self, parents, school principal, teachers) measures, longitudinal	Path Modelling	12
Espelage et al. (2016)	USA	*n* = 482 (65% female, 35% male)	Undergraduate students (M = 19.98)	Survey of childhood bullying victimisation, polyvictimisation and psychological functioning	Correlational Analyses; Regression	11
Isaacs et al. (2008)	Finland	*n* = 177 (57.1% female, 42.9% male)	14–15 → 22–23 years	Peer nominations in adolescence, administered questionnaires during school hours; mailed questionnaires in young adulthood	Regression; Moderation	9.5
Iyer-Eimerbrink and Jensen-Campbell (2019)	USA (Texas; school-based sample)	*n* = 120 (55% female, 45% male)	12–17 years	Self- and parent-reported measures of victimisation, psychological and health	Regression	10.5
Låftman et al. (2024)	Sweden	*n* = 2323 (~58% female, ~42% male)	15–16 → 17–18 → 20–21 years	Self-report surveys	Logistic Regression	10.5
Lee et al. (2024)	Burkina Faso	*n* = 1160 (40.4% female, 59.6% male)	12–20 years	Standardised interviews, validated scales	Logistic and Negative Binomial Regression	9
Lereya et al. (2015)	UK (Avon) and USA (North Carolina)	ALSPAC *n* ≈ 4026; GSMS *n* ≈ 1420 (Gender proportions vary by cohort)	8–13 years	Parent/child reports (ALSPAC); interviews (GSMS); linked follow-ups	Logistic Regression	11.5
Liu et al. (2020)	China (East China)	*n* = 879 (42% female, 58% male)	12–15 years	Self-report surveys on cyberbullying, rumination, depression, PTSS	Mediation	10.5
Lund et al. (2009)	Denmark	*n* = 6094 men (male-only cohort)	Any school-age	2004 questionnaire; registry linkage; self-report recall of bullying and depression	Logistic and Multivariate Logistic Regression	10
Manrique et al. (2020)	Sweden (national cohort Futura01)	*n* = 270 (76.7% female, 33.3% male)	15–16 → 17–18 → 20–21	Self-report surveys (bullying victimisation and depression), across three waves	Logistic Regression	10.5
Nania et al. (2024)	Canada (Western Canada)	*n* = 182 (72% female, 28% male)	10–18 years	Baseline and 3-month follow-up surveys; pain program cohort	Mediation	9
Orengul et al. (2023)	Turkey (Istanbul)	*n* = 70 (~50% female, ~50% male)	7–10 years	Screening with TDBRS, psychiatric interviews, follow-up surveys	Group Comparisons; Regression	9.5
Perren et al. (2013)	USA	*n* = 478 (49.8% female, 50.2% male)	10–13 years	Child, parent, teacher questionnaires; peer reports; attribution scenarios	Mediation; Moderation	9.5
Rudolph et al. (2011)	USA	*n* = 433 (~55% female, ~45% male)	7–11 years	Repeated child self-reports and teacher reports of peer victimisation; mental health questionnaires	Latent Growth Curve Models	9.5
Turner et al. (2020)	USA	*n* = 791 (gender distribution not specified)	8–17 years	Youth self-report surveys (Crimes Against Children Research Centre)	Trajectory Analysis; Regression	10
Winding et al. (2020)	Denmark (West Jutland)	*n* = 1790 (gender distribution not specified)	15–18 years	Self-report surveys in 2004, 2007, 2017	Multiple Logistic Regression	11
Wright and Wachs (2019)	USA	*n* = 416 (~46% female, ~54% male)	12–13 years	Self-report Questionnaires	Hierarchical Regression; Moderation	10.5
Wright et al. (2022)	USA	*n* = 466 (52% female, 45% male, 2% other)	14–16 years	Self-reports questionnaires	Structural Regression model	10

**Table 2:** Summary of Studies’ Main Findings.

Study	Type and Measurement of Peer Victimisation	Mental Health Outcome	Time from Victimisation Exposure	Main Test Statistics	Test Name	Variables Controlled for	Mediators	Moderators
Bouffard and Koeppel (2014)	Self-reported repeated bullying before age 12	General Negative Mental Health (emotional/mental health problems)	6–9 years	b = 0.509, *p* < 0.01	OLS regression	Age, gender, race, marital status, household income; medication for chronic condition or learning disability	/	/
Boyes et al. (2014)	Social and Health Assessment Peer Victimisation Scale (9 items; adapted from MPVS); self-report via interviewer-administered survey	Internalising problems (anxiety, depression, PTSD symptoms)	1 year	β = 0.04, *p* < 0.05	Cross-lagged path models	Age, gender, poverty, urban/rural, baseline internalising scores	/	/
Boyes et al. (2020)	Social and Health Assessment (SAHA) Peer Victimisation Scale, 9 items; self-report (interviewer- or tablet-assisted)	Depression (D) Anxiety (A) PTSD (P)	18 months	**D**: β = 0.05, *p* = 0.165 **A**: β = 0.01, *p* = 0.780 **P**: β = −0.01, *p* = 0.722	Path model	Baseline depression, anxiety and PTSD, age, gender, poverty, urban/rural	* Internalised HIV stigma: **D**: β = 0.01, *p* = 0.048 **A**: β = 0.01, *p* = 0.030 **P**: β = 0.02, *p* = 0.049	/
Copeland et al. (2013)	Victimisation or bullying reported by child/parent	Anxiety disorders (A) Depression (D) Suicidality (S)	~10 years	* **A**: OR 4.3 (2.1–8.6) * **D**: OR 2.3 (0.8–6.2) * **S**: OR 1.2 (0.4–3.3)	Weighted logistic regression with GEE	/	/	/
Cosma et al. (2018)	Romanian version of the Bullying Questionnaire (Olweus & Hart, 1993)	Internalising Problems	6 (T2) and 12 (T3) months	T1 → T2: B = 0.042, SE = 0.197, *p* = 0.832 T2 → T3: B = 0.212, SE = 0.206, *p* = 0.304	Cross-lagged panel SEM	/	/	/
Due et al. (2009)	Self-reported single item: “Were you bullied at school?”	Depression	~12 years	*p* = 0.0016	General Linear Model (ANOVA)	Sex and SES	/	Bullying-depression association stronger in low SES
D’Urso et al. (2024)	Self-reported single item: “Have you been bullied in the last 3 months?”	Internalising problems; emotional well-being	4 years and 7 years	B = 0.088, SE = 0.03, *p* < 0.01 (17 years); B = −0.064, SE = 0.03 (20 years)	Path modelling	Gender, social class, and cognitive ability	/	/
Espelage et al. (2016)	University of Illinois Victimisation Scale (adapted to cover entire childhood) + added cyber item	Depression (D) Anxiety (A) PTSD	A few years: from childhood to early 20s	**D**: β = 0.19; *p* < 0.001; R^2^ = 0.13 **A**: β = 0.12; *p* < 0.01; R^2^ = 0.10 **PTSD**: β = 0.29; *p* < 0.001; R^2^ = 0.21	Linear regression	Gender, community violence	/	/
Isaacs et al. (2008)	Peer nomination of victims and self-reported victimisation	Self-esteem (SE) Depression (D)	~ 8 years	* **SE**: r *= −0.18*, *p* < 0.05 * **D**: r *= 0.26*, *p* < 0.01	Hybrid longitudinal path model	Baseline self-esteem and depression	/	* Family support
Iyer-Eimerbrink and Jensen-Campbell (2019)	Direct and Indirect Aggression Scales	Anxious Depression (AD) Withdrawn Depression (WD) PTSD	~ 24.5 months	* AD: b = 0.23 (0.05– 0.41); *p* < 0.05 **WD**: b = 0.10; (−0.08– 0.29); *p* = 0.31 * PTSD: B = 0.33 0.16–0.50); *p* < 0.001	Linear regressions	Gender	/	/
Låftman et al. (2024)	Self-reported single item	Depression symptoms (D) Anxiety symptoms (A)	~ 3–6 years	Males: * **D**: OR 2.67 (0.88–8.09); **A**: OR 0.37 (0.05–2.85) Females: * **D**: OR 2.40 (1.18–4.86); *A: OR 2.37 (1.18–4.77)	Binary logistic regressions	Family type, parental education and country of birth, prior medication for depression/anxiety	/	Gender (ns)
Lee et al. (2024)	Self-report interview and one self-report item	Depression (D) PTSD Self-harm (SH)	1 year	* D: b = 0.99 (0.82–1.19) * PTSD: b = 1.89 (1.13–3.17) * SH: b = 1.37 (0.77–2.45)	Weighted negative binomial regression	Age, household wealth quintile, physical fighting in the past 12 months, and baseline mental health	/	* Gender: **D:** F(7,376) = 3.43, *p* = 0.001; **P**: F(7,376) = 3.47, *p* = 0.001 **SH**: F(7,376) = 1.12, *p* = 0.35)
Lereya et al. (2015)	Child/parent-reported Bullying and Friendship Interview Schedule	Anxiety (A) Depression (D) Self-harm/suicidality (SS)	Two Cohorts: ALSPAC: ~ 5–10 years GSMS: ~ 3–10 years	ALSPAC: *A: OR 1.7 (1.4–2.2); * **DD**: OR 2.3 (1.8–3.0); * SS: OR 1.7 (1.4–2.2) GSMS: * **A**: OR 4.9 (2.3–10.4); * **DD**: OR 4.7 (2.5–8.9); * **SS**: OR 3.0 (1.2–7.7)	Linear regression	Sex, socioeconomic status, family instability and family dysfunction	/	/
Liu et al. (2020)	Self-reported Revised Cyber Bullying Inventory—Cyberbullying Subscale	Depression (D) PTSS	8 months	* D: β = 0.39, *p* < 0.001 * PTSS: β = 0.27, *p* < 0.001	Mediation models	Age, gender, grade, Internet use (time and frequency)	* Intrusive rumination * Deliberate Rumination	/
Lund et al. (2009)	Self-reported single item (retrospective)	Depression	~45–35 years	Bullied vs. never bullied: OR 1.21 (0.88–1.66)	Linear regression	Parental mental health, adult social class	/	/
Manrique et al. (2020)	Adolescent Peer Relations Instrument victimisation items	Depression (D) Anxiety (A) PTSD	A few years earlier	**D**: Physical β = 0.20, *p* < 0.01; Verbal β = 0.35, *p* < 0.001; Social β = 0.29, *p* < 0.001. Model R^2^ = 0.20–0.28 across bullying types. **A**: Physical β = 0.32, *p* < 0.001; Verbal β = 0.3, *p* < 0.001; Social β = 0.35, *p* < 0.001. Model R^2^ = 0.13–0.17. **PTSD**: Physical β = 0.24, *p* < 0.001; Verbal β = 0.32, *p* < 0.001; Social β = 0.24, *p* < 0.001. Model R^2^ = 0.22–0.26	Hierarchical linear regressions	Sex and race	Perceived social support partially mediated bullying → depression and bullying → PTSD; No significant mediation for anxiety.	
Nania et al. (2024)	Self-reported Social Experience Questionnaire	PTSS	3 months	* **PTSS**: b = 1.17 (0.47–1.87), t = 3.30, *p* = 0.001	Mediation model (PTSS mediator; direct path victimisation -> PTSS extracted)	Age and gender	/	/
Orengul et al. (2023)	Self-reported Revised Olweus Bully/Victim Questionnaire	Internalising difficulties (ID) Anxiety (A)	1 year	**ID**: b = 0.27; t = 1.96; *p* = 0.054	Linear regression	/	/	/
Perren et al. (2013)	Peer nominations of victims	Internalising difficulties (DP)	~2 years	* **ID**: B = 0.20, *p* < 0.001	SEM	Gender	Hostile attributions partially mediated T1 victimisation → T3 externalising (≈13% of total effect)	* Self-blame: b = 0.03, *p* < 0.01 and ~+ 0.04 residual change of slope per +1 unit victimisation
Rudolph et al. (2011)	Revised Social Experiences Questionnaire; child and teacher report	Depressive symptoms (D)	~3 years	* D: β = 0.31, *p* < 0.01 (boys) β = 0.39, *p* < 0.001 (girls)	Latent growth curve analysis	Baseline depressive symptoms	/	Sex non-significant for depressive symptoms
Turner et al. (2020)	JVQ peer items (physical assault, physical intimidation, emotional bullying, dating violence) and Internet harassment item. Self-report via telephone interview	Trauma symptoms	~2 years	b = 0.99 (0.40–1.57)	Linear regression	Age, sex, race/ethnicity, SES, family structure	/	/
Winding et al. (2020)	Self-reported single item	Depression	10–13 years (victimisation assessed at 15 and 18 years)	* Age 15 bullied: OR 1.6 (1.2–2.0) * Age 18 bullied: OR 2.1 (1.4–3.0) * One age point: OR 1.8 (1.3–2.3) * Two age points: OR 2.7 (1.5–4.8)	Multiple logistic regression	Gender, parental education, split home, close friends, family functioning	/	/
Wright and Wachs (2019)	Self-report cyber victimisation scale	Depression (D) Anxiety (A) Loneliness (L)	1 year	* **D**: β = 0.21, *p* < 0.01 * **A**: β = 0.18, *p* < 0.05 * **L**: β = 0.22, *p* < 0.05	Hierarchical multiple regression	Gender, ethnicity, face-to-face victimisation, and baseline mental health	/	School belongingness: β = 0.16, *p* < 0.05 (D), β = 0.13, *p* < 0.05 (A), β = 0.15, *p* < 0.05 (L)
Wright et al. (2022)	Self-reported cyber victimisation (9 items)	Depression (D) Anxiety (A)	1 year	* **D**: β = 0.32, *p* < 0.001 * **A**: β = 0.27, *p* < 0.001	Structural regression	Gender and baseline mental health	/	Perceived social support: β = −0.13, *p* < 0.05 (D); β = −0.16, *p* < 0.05 (A)

* Significant predictors (*p* < 0.05).

## Data Availability

Data supporting the results of this study are accessible from the corresponding author upon request.

## Appendix A

**Full database search strategies**

**Scopus:**

TITLE-ABS-KEY ((bully* OR “peer victim*” OR “peer aggression” OR “cyberbully*” OR “peer harassment”) AND (“post-traumatic stress” OR “posttraumatic stress” OR PTSD OR “PTSS” OR “trauma symptom*” OR “traumatic stress” OR “psychological consequences” OR “long-term consequences” OR “mental health consequences”) AND (child* OR adolescent* OR teen* OR youth) AND (longitudinal OR prospective OR “follow-up” OR “long term” OR chronic OR sustained))

**Embase:**

(‘bullying’/exp OR bully*:ti,ab OR ‘peer victim*’:ti,ab OR ‘peer aggression’:ti,ab OR ‘school violence’:ti,ab OR cyberbully*:ti,ab OR ‘peer harassment’:ti,ab) AND (‘posttraumatic stress disorder’/exp OR ‘post-traumatic stress’:ti,ab OR ‘posttraumatic stress’:ti,ab OR PTSD:ti,ab OR PTSS:ti,ab OR ‘trauma symptom*’:ti,ab OR ‘traumatic stress’:ti,ab OR ‘psychological consequences’:ti,ab OR ‘long-term consequences’:ti,ab OR ‘mental health consequences’:ti,ab) AND (child*/exp OR child*:ti,ab OR adolescent*/exp OR adolescent*:ti,ab OR teen*:ti,ab OR ‘young adult*’:ti,ab OR youth:ti,ab OR student*:ti,ab) AND (longitudinal:ti,ab OR prospective:ti,ab OR cohort:ti,ab OR ‘follow-up’:ti,ab OR ‘long term’:ti,ab OR chronic:ti,ab OR persistence:ti,ab OR sustained:ti,ab)

**PubMed:**

((“Bullying”[MeSH] OR bully*[tiab] OR “peer victimization”[tiab] OR “peer victimisation”[tiab] OR “peer victim*”[tiab] OR “school bullying”[tiab] OR cyberbully*[tiab]) AND (“Stress Disorders, Post-Traumatic”[MeSH] OR PTSD[tiab] OR “posttraumatic stress”[tiab] OR “post-traumatic stress”[tiab] OR “trauma”[tiab] OR “traumatic stress”[tiab] OR “complex PTSD”[tiab] OR “post traumatic”[tiab] OR “psychological consequences”[tiab] OR “long-term consequences”[tiab] OR “mental health consequences”[tiab]) AND (child[MeSH] OR adolescent[MeSH] OR teen*[tiab] OR child*[tiab] OR adolescen*[tiab]))

**ProQuest:**

(ti(bully* OR “peer victim*” OR “peer aggression” OR cyberbully* OR “peer harassment”) OR ab(bully* OR “peer victim*” OR “peer aggression” OR cyberbully* OR “peer harassment”) OR su(bully* OR “peer victim*” OR “peer aggression” OR cyberbully* OR “peer harassment”)) AND (ti(“post-traumatic stress” OR “posttraumatic stress” OR PTSD OR PTSS OR “trauma symptom*” OR “traumatic stress” OR “psychological consequences” OR “long-term consequences” OR “mental health consequences”) OR ab(“post-traumatic stress” OR “posttraumatic stress” OR PTSD OR PTSS OR “trauma symptom*” OR “traumatic stress” OR “psychological consequences” OR “long-term consequences” OR “mental health consequences”) OR su(“post-traumatic stress” OR “posttraumatic stress” OR PTSD OR PTSS OR “trauma symptom*” OR “traumatic stress” OR “psychological consequences” OR “long-term consequences” OR “mental health consequences”)) AND (ti(child* OR adolescent* OR teen* OR youth) OR ab(child* OR adolescent* OR teen* OR youth) OR su(child* OR adolescent* OR teen* OR youth)) AND (ti(longitudinal OR prospective OR “follow-up” OR “long term” OR chronic OR sustained) OR ab(longitudinal OR prospective OR “follow-up” OR “long term” OR chronic OR sustained) OR su(longitudinal OR prospective OR “follow-up” OR “long term” OR chronic OR sustained))
